# Which Factors Drive Consumer Decisions during Milk Purchase? New Individuals’ Profiles Considering Fresh Pasteurized and UHT Treated Milk

**DOI:** 10.3390/foods11010077

**Published:** 2021-12-29

**Authors:** Valentina Maria Merlino, Stefano Massaglia, Danielle Borra, Antonio Mimosi, Paolo Cornale

**Affiliations:** Department of Agricultural, Forest and Food Sciences, University of Turin, Largo Paolo Braccini 2, 10095 Grugliasco, Italy; valentina.merlino@unito.it (V.M.M.); stefano.massaglia@unito.it (S.M.); danielle.borra@unito.it (D.B.); antonio.mimosi@unito.it (A.M.)

**Keywords:** cow milk, fresh pasteurized, ultra-high temperature (UHT), best-worst scaling, socio-demographic profiles

## Abstract

The cow’s milk market is going through a critical period characterised by a continuous contraction in consumption as a consequence of the lack of competitiveness on the market of the conventional product (commodity) versus numerous specialties. This paper aimed to define the profiles of milk consumers in terms of individual preferences (assessed using the best-worst scaling methodology) and socio-demographic features. A survey was conducted in several stores of large-scale retail, convenience stores, and open-air markets distributed in north-west Italy to collect data from 1216 respondents. For milk shopper purchasing habits, two consumer groups were defined and compared in terms of preferences: the fresh pasteurized milk consumer (FPc) (56% of the total sample) and the ultra-high temperature treated milk consumer (UHT_c_) (35%). A series of two-ways multivariate analysis of variance (MANOVA) were conducted to assess the effect of individuals’ socio-demographic characteristics and the type of milk chosen on the consumer preferences, simultaneously. Significant differences in milk purchasing habits and preferences emerged when comparing the two consumer groups (UHT_c_ and FP_c_). Empirical evidence of the study supported the starting hypothesis, suggesting the significance or relevance of the consumer socio-demographic characteristic, as well as their interaction effect with the type of milk on the level of importance given to the considered milk quality attributes. On the contrary, the gender results were not significant for the milk preferences definition. The assessment of consumer preferences, associated with the individuals’ socio-demographic characteristics could have important implications for outlining more effective marketing strategies based on a more targeted communication (i.e., related to the sustainability dimension of the local product, nutritional value and brand), leading the consumer back to the commodity rediscovery concerning individuals’ features and habits.

## 1. Introduction

The influences of globalisation on eating habits have led to an increase in the variety of diets by including new foods, ingredients, and preparations. At the same time, the growing influence of values, beliefs, and norms (ethical, health or sustainability-related) in defining individuals’ attitudes and preferences and purchasing behaviour has led to important changes in consumers’ diet composition [1,2,3,4]. In particular, this trend is very impactful in the European community, as reported also by recent data published by Eurobarometer (80% of European citizens buy sustainable products) [5]. This evolution has prompted consumers to include new foods and exclude others [6,7,8,9]. Among the latter is bovine milk. Milk has always been considered a fundamental component of human nutrition; it is healthy, beneficial, and fortifying for all age groups. Cow’s milk is included in one of the seven basic food groups developed through the collaboration between the National Research Institute for Food and Nutrition (INRAN) and the Italian Society of Human Nutrition (SINU) [10] for providing high biological value proteins, calcium, and phosphorus, as well as being a relevant source of bioactive components (e.g., immunoglobulins, conjugated linoleic acid, lactoferrin, etc.) with beneficial effects on human health [11]. Even considering the negative effects on human health from excessive milk consumption, the essentiality of milk in human nutrition in a balanced diet has been demonstrated in several researches [12,13,14,15]. However, this product is no longer part of the consumers’ dietary plan [16,17], especially in some countries of the world. 

### 1.1. The Recent Drinking Cow Milk Market Trends: Consumption and Supply Orientations 

Claims concerning cow milk’s negative effects on human health [13], the impact on the environment associated with its production [18,19,20], the perception of poor welfare experienced by dairy cows kept in intensive farming system [21], as well as the new eating styles of the population (hyper-proteinic, keratogenic, or health-conscious) [22] probably contributed to a continuous contraction in milk consumption. However, in a countertrend, “green” consumption orientations are emerging, characterized by food choices of products, including milk, which are linked to tradition and are local and sustainable [23]. The dairy sector in Italy is extremely heterogeneous in terms of territorial distribution, companies’ size, and herd management, with a good proportion of small family-run businesses based on traditional production systems [24] that are more sustainable for the environment and the territory in which they are located [25]. It is therefore important to properly communicate the value (e.g., local branding) and the higher quality of the products obtained from these local systems as differentiation tools on the market to increase transparency and create greater consumer awareness [1,26,27]. Approaches in communicating product values and quality to the consumer include: (i) brand or seller reputation [28,29,30,31], (ii) quality assurance related to the origin of the product [32,33,34], and (iii) intrinsic characteristics (e.g., taste, nutritional value, price, environmental sustainability, etc.) [27,35,36,37,38]. However, targeted and attractive communication campaigns need to be carefully designed and developed to cope with the high level of competition between the many brands in the dairy market. The offer composition of drinking milk in the large-scale retail trade in Italy, as shown in Merlino et al. [39], is extremely varied, showing differences in terms of product types, milk origin, prices, and brands, but balanced in terms of the composition of references between fresh pasteurized (FP) and ultra-high temperature (UHT) processed milks. In parallel, the milk supply is also characterized by a high competition from the specialties products (milks with added vitamins, mineral salts, Omega 3 fatty acids, etc.) that reached a considerable level of detail in last years. However, the addition of beneficial substances prefigures in the milk sector the creation of denaturalised products, which move away from the most common ideal of the milk/tradition combination. This trend could risk being reduced to small market niches, with limits in terms of business that this entails, following the market dynamics relating to the emphasis on sub-segmentation [40,41]. Large competitors that operate in large volumes are required to balance the demand of products segmentation with the need to stay on course towards the marketing of milk suitable for mass consumption (with benefits that may be specific but meet widespread nutritional needs). In this regard, it remains of fundamental importance to understand if, how much, and how consumers orient themselves and choose the two major product categories of cow’s milk, namely FP and UHT treated milk. According to Ding and Veeman [30], consumers of FP milk are loyal to a known brand rather than to certification, focusing their purchases on products in which they recognise high levels of trust as guarantee of milk safety and healthy. Consumers also connect FP milk with local production, short chain, tradition as well as high nutritional value and good taste [30,42,43,44]. Conversely, although UHT milk has lower nutritional value, consumers also recognize to it important characteristics (e.g., storability and a lower price per litre) that have determined its success in the domestic market in recent years [45,46,47]. In addition, it is demonstrated that socio-demographic (SD) and personal variables always drive consumer choice and preference creation [6,48,49,50].

#### The Italian Milk Market: Fresh Pasteurized vs. Ultrahigh Temperature (UHT) Treated Milk 

Italy is one of the leading milk producers in Europe, with about 12.5 million tons produced in 2019 (ISMEA, Roma, Italy, 2020). In 2019, the production of drinking milk in Italy was equal to 1.4 million tons, of which about 60% is UHT processed milk, while FP milk accounted for the remaining 40% [17]. Drinking milk has lost about 18% of its sales in the last five years, mainly affecting FP milk, whose per capita consumption has declined by 25–30% in the same period [51]. This negative consumption trend has also affected UHT milk. Companies’ reaction has been to focus on premium niches and on types with greater added and perceived value. Lactose-free milk has a 17% share of total market turnover, with sales continuing to grow (+35% over the five-year period). Organic milk is also gaining increasing acceptance among consumers nationwide, with growth rates of 32.8% over the last five years [52]. However, in the previous two years, it seems that in some areas, the consumption of “traditional” milk is restarting driven by product sustainability and local production [53], as well as to the content of specific nutrients with beneficial effects on health [51,54]. In fact, it seems that milk consumption is going through a vital phase, particularly the UHT type (+10% in 2020 with respect to the previous year) [55]. The biggest competitor to UHT milk is fresh milk. It shares similarities as strong as the distances between the two sectors, one belonging to the group of long-life products and the other to perishable products [56]. However, FP milk consumption maintains negative trends in Italy (−5.5% in 2020 with respect to the previous year) [55].

### 1.2. Research Aims Definition

This research aims at answering the following questions: (i) What are the attributes of choice for UHT and FP milk consumers? (ii) Are there statistically significant differences in milk quality attributes perception based on socio-demographic variables and type of milk?

Even though market data show signs of recovery in milk consumption, there is a lack in the literature for recent research projects analysing the perception, preferences, and consumption orientations towards cow’s milk. Furthermore, while there are several works on the assessment of consumer preferences towards milk, few studies are based on new methodological approaches that solve some of the weaknesses of traditional methodologies used in consumer research, such as discrete choice methodologies. In this regard, the limited understanding of consumer preferences and characteristics represents a critical limitation in product development and relaunch. At this purpose, the present research was based on a choice experiment directly involving a milk consumer sample of north-west Italy by developing the design of the best-worst scaling (BWS) methodology. The study was conducted to assess the level of preferences of consumers towards a set of quality attributes that describe the product [57,58] differentiating the of FP and UHT treated milk consumers (FP_c_ and UHT_c_, respectively). 

Starting with a choice experiment involving a sample of individuals, the results of this approach provide a quantitative indication of preference for each attribute submitted to the sample. Although it finds increasing confirmation in the literature, including in the agri-food sector [33,50,59,60], to the best of our knowledge, it has been little used in studies on cow’s milk consumers [61,62] and, in particular, in the Italian context. During the choice experiment, respondents repeatedly evaluate a predefined set of items describing the product by choosing among them what they rate as the best and worst for selecting the product [57]. Moreover, this methodology enables exploring various attributes of different nature simultaneously in the same choice experiment. This latter aspect, in our opinion, leads to the best condition for studying preferences, as it projects the consumer into a more realistic situation that can be associated with the complexity of choices made in real life [63]. In addition, the BWS allows nominal information to be processed in the same model with numerical information, thus allowing to combine preference information (numerical preference index) with qualitative SD details of the consumer. This methodological approach allows to describe the consumer profiles in terms of preferences and socio-demographic characteristics filling a gap in the scientific literature related to the study of milk consumers, especially in the Italian context. For this purpose, in addition, the effect of SD characteristics and the type of milk (FP or UHT) on the consumer preferences was also analysed to test the following hypothesis:

**Hypothesis** **1** **(H1).** *There are significant differences of consumer preferences towards milk attributes influenced by individuals’ socio-demographic characteristics and their choice concerning milk type.*

The paper is organised as follows: in Section 1.1, we briefly introduce the Italian milk market and the current consumption trends; in Section 1.2, we define the aims definition scheme. Section 2 describes materials and methods, while the empirical results and discussion are given in Section 3. Finally, the conclusions are noted in Section 4.

## 2. Materials and Methods

### 2.1. Questionnaire Development and Data Collection

A choice experiment based on face-to-face interviews was conducted in several stores of large-scale retail, convenience stores, and open-air markets distributed in north-west Italy to investigate consumers’ stated preferences towards the intrinsic-extrinsic and credence attributes of cow’s milk. The surveys were conducted using a paper questionnaire from April to August 2019, from Monday to Sunday, in two-time slots (from 8 to 12 a.m. and from 3 to 8 p.m.), randomly intercepting respondents outside the stores or, in the case of the markets, among the crowd of shoppers. Participation of all respondents was voluntary, and all respondents provided informed consent. The survey was conducted following the ethical standards set out in the Declaration of Helsinki. The questionnaire was developed in Italian and approved by the University Bioethics Committee of the University of Turin (https://www.unito.it/ricerca/strutture-e-organi-la-ricerca/comitato-di-bioetica-dellateneo/ (accessed on 1 December 2021). The questionnaire was structured in three main sections described in Table 1, alternating closed-ended and multiple check-all-that-apply (CATA) questions (that allows to mark out as many options as are needed to express their more relevant options/preferences) (Section 1 and Section 2), the latter used to investigate on individual’s milk purchasing and consumption habits [64]. The final part of the questionnaire (Section 3) was devoted to detecting consumer preferences according to the Best-Worst scaling methodology experimental scheme. Coming to the last section, respondents were asked to choose, for each subset of milk attributes, the most important (BEST) and least important (WORST) item for their choice of milk. Starting with a selection of 12 milk attributes, the BWS experimental design adopted in our research was developed using Sawtooth MaxDiff Designer software (SSI-version 8.4.6, Sawtooth Software, Orem, UT, USA; http://www.sawtoothsoftware.com/ (accessed on 1 December 2021) as previously describe in Tabacco et al. [65]. The 12 attributes of milk were selected based on other research selected from papers published from 2010 to 2020 in ISI or Scopus indexed journals on consumer food preferences assessment and milk in particular [6,54,66,67,68,69,70,71,72,73]. Selected items were intrinsic (fat content-skim, partially skimmed, or whole-expiration date, taste, nutritional value), extrinsic (price, packaging material-plastic bottle, carton, glass-brand, information in the label), and belief attributes (organic certification, high-quality certification, local origin, country of origin). 

### 2.2. Data Analysis 

During the choice experiment, the individual who expresses his preference will select the two alternatives belonging to a specific choice set, ***y*** and ***j***, to guarantee the maximum possible utility. Considering this assumption, the level of preference for single attribute is proportional to the frequency with which the single respondent has chosen it as BEST and WORST, according to the approach of the random utility theory (RUT) [74] at the basis of the paired comparison method from which the BWS [75] is developed. The RUT assumes that an individual (***i***) provides a level of utility to an alternative (***ay***) evaluated in a set of alternatives, which can be described by the following formula:

***ay* = *Ua_i_* + *εsa_i_*** (1)

where ***Ua*** is the measurable and directly observable systematic component, while **εs** represents the model error.

The total utility for a product of the term ***Ua***, provided by individual ***i***, is influenced by the characteristics of the product (***β***) (for each individual ***i***, in ***n*** cases) and is defined in the following equation:

(2) $$Uai=\overset{N}{\underset{n=1}{\sum }}\left(\beta_{in}+X_{inq}\right)$$

where the total utility of the alternative is the sum of the partial utilities of each level for each attribute (***N*** attributes) (de-Magistris et al., 2017). Assuming that the choice of the i-th consumer depends on the additional utility (***Z_i_***) resulting from the purchase of product a with respect to product n, the latter can be expressed as follows:

(3) $$Z_{i}=U_{ia}-U_{in}=\left(V_{ia}+\epsilon_{ij}\right)-\left(V_{in}+\epsilon_{in}\right)=\left(V_{ia}-V_{in}\right)+\left(\epsilon_{ia}-\epsilon_{in}\right)$$

It is assumed that the individual for each set of choice identifies the difference in utility for each pair of attributes a and b (*Uiai-Uib*), identifying the one with the most remarkable difference in utility between the labels. If a choice set has m items, then there are m(m − 1) possible BEST-WORST combinations that a person could choose. The particular pair of items selected by the consumer as best and worst (the one with the maximum difference in importance), therefore, represents a choice among all possible m(m − 1) pairs [76]. This methodology provides as output the average utility per item calculated as a function of the responses of a sample of individuals with changing numerosity. In this research, this utility level (preference level) was calculated from the sample responses using Sawtooth software (SSI version 8.4.6, Orem, UT, USA; http://www.sawtoothsoftware.com/ (accessed on 1 December 2021)). Feedback collected from the interviews was analysed to obtain the count report and hierarchical Bayes estimation (HB) for stated preferences assessment. The count report provides for each attribute the number of times it was selected as best (COUNTbest) and the number of times it was selected as worst (COUNTworst). In the HB report, the aggregation of the responses from all respondents estimates the single attribute level scores useful to define an ordered scale of attributes based on a quantitative value calculated according to the average preferences level declared by the respondents (Average Raw score or A-RS). In particular, this is defined by the difference between COUNbest and COUNTworst, related to the sample size multiplied by the number of times the single attribute appears in the questionnaire (r = 3). This raw index can have a positive and negative value, and the sum of all aggregate scores makes 0. Values below 0 indicate that that attribute was chosen fewer times as the preferred attribute for the choice of milk. At the same time, the higher preference score is linked to the most important attribute for the involved consumer sample [77]. The software estimates the HB model using a Monte Carlo Markov Chain algorithm [78]. The HB method combines a priori distributions of variables with individual choice data in order to estimate a posteriori distributions for each involved subject [79,80,81]. The standard deviation was used as a raw indicator of the variability present within the sample. The A-RSs were calculated considering the responses to all milk attributes to create two different individual-level scales obtained from individuals belonging to two consumer groups [82]: the FP_c_ and the UHT_c_. These latter were created considering the answers received to question 2a (“What type of cow’s milk do you buy?”) (Table 1) and allocating the individuals who had declared to choose only FP milk in the group FPc, and the purchasers of only UHT milk in the group UHT_c_. The significant differences among the SD variables between the two consumer groups were assessed using the non-parametric statistic Chi-square test. The non-parametric test Mann-Whitney U was performed to assess the statistically significant differences between the mean value of preferences (A-RSs) expressed for each milk attribute towards the two consumer groups (FP_c_ and UHT_c_). The individuals that purchased both the milk types were excluded from the analysis. To test H1 (there are significant differences of consumer preferences towards milk attributes influenced by individuals’ SD characteristics and their milk choice-fresh pasteurised or UHT), the Multivariate analysis of variance (MANOVA) was employed [48,83]. We performed six two-ways MANOVAs in order to test the main and the interaction effects of the type of milk (two levels: FP and UHT) with each of the other independent variables (gender, age, family size, economic situation, occupation and educational level) on the consumer preferences to the 12 considered milk attributes (dependent variables).

All of the non-parametric tests and the MANOVA analysis were performed in SPSS for Windows, version 27.0 (SPSS Inc., Chicago, IL, USA). Finally, the characteristics of the two consumer profiles collected by Section 2 of the questionnaire were qualitatively described to define purchasing and consumption habits of FP and UHT consumers.

## 3. Results and Discussion

### 3.1. Consumer Sample Composition

A total of 1216 individuals were interviewed during the data collection phase. Considering the respondents’ answers to question 2a (“What type of cow’s milk do you buy?”), 677 consumers (that only buy FP milk) were grouped in the FPc and 430 (that only buy UHT milk) in the UHT_c_ groups. On the contrary, 105 individuals who declared to purchase both the product types (FP and UHT milk) were excluded by the analysis. The SD characteristics of the total consumer sample and of FP and UHT consumers are described in Table 2. Comparing the two groups, they differ significantly considering all the socio-demographic variables, except for gender.

In general, the SD characteristics of the whole consumer sample were representative of the national population of cow’s milk buyers according to Nielsen surveys [84] and comparable with those found in other national research on milk consumers [85,86,87].

### 3.2. Effect of Milk Consumer Choices (Type of Milk) on Individual Preferences as Well as on Purchasing and Consumption Habits

**Figure 1:**
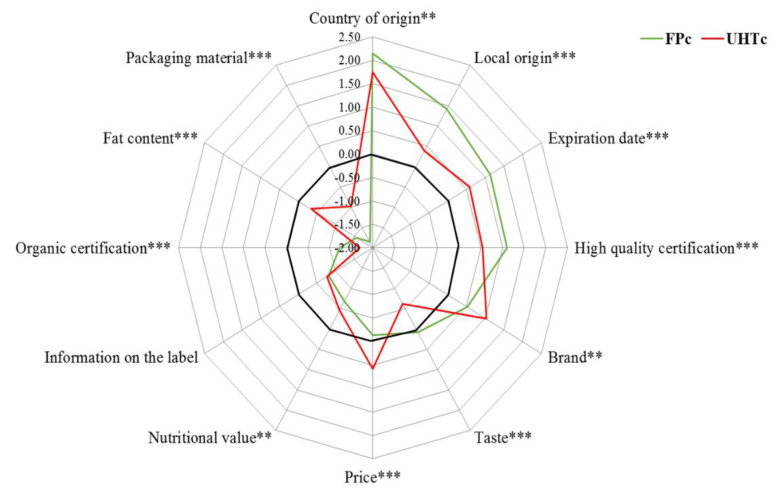
Level of importance (average raw scores) given by the FP and UHT milk consumers to the 12 milk quality attributes. Notes: the differences between the average raw scores for each milk attribute between the two consumers’ groups tested through the Mann-Whitney U test were significant at the 5% (**) and at the 1% level (***).

The differences among the A-RSs calculated for each considered milk attribute are reported in Figure 1 comparing the FP_c_ and UHT_c_ preferences.

**Figure 2:**
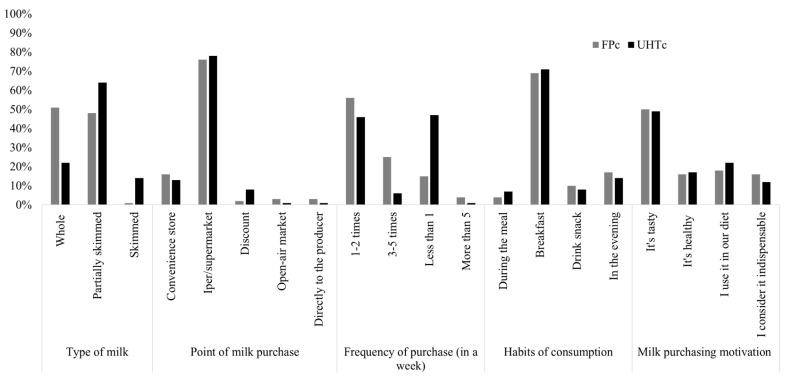
Milk purchasing and consumption habits of the two consumer groups: fresh pasteurized (FP_c_) and UHT milk consumers (UHT_c_).

In addition, the description of milk consumers purchasing and consumption habits are reported in Figure 2 comparing FP and UHT consumers.

In general, the level of importance given to the selected milk attributes differs significantly considering all cases, except for the attribute “label information” that both profiles consider unimportant for milk choice (Figure 1). While consumers of fresh milk choose semi-skimmed and whole milk equally, consumers of UHT milk mainly choose semi-skimmed (Figure 2). The choice of milk store, as well as the consumption habits and motivations, are comparable between the two groups of consumers. In particular, both targets buy milk mainly from large retail chains, to be consumed during breakfast, and due to the fact that it tastes good. On the contrary, the two groups differ in the frequency of weekly milk purchase as FP_c_ buy it two to five times a week, while UHT_c_ consumers are divided between those who buy it one to two times and those who buy it less than one time a week.

Focusing on the preference degree given by the UHT milk consumers (*n* = 430), this group choice of milk primarily assesses the product origin, followed by expiry date, brand, and price. These results agree with previous studies on the assessment of UHT milk consumer preferences at the European level, in which there emerges a relevance of expiry date, price, and brand in the decision-making process [88,89]. In addition, the importance of price is often associated with the choice of utility products, such as processed UHT milk, as also shown in Maehle et al. [90]. However, in contrast to other research, our results underline a focus by milk consumers on the importance of product origin recognition. This result, especially in UHT milk, can lead to different interpretations, such as the individual search for safety, familiarity, traceability, and transparency on indigenous-originated agri-food products [91,92,93]. Many studies have confirmed that consumers are willing to pay a premium price for a national and local food product [93,94,95]. However, the recurring issue in UHT milk is the generic indication of milk origin, which allows producers to indicate “EU country” or “no-EU” as an indication of origin on the label. Producers can specify, but on a voluntary basis only, the country of origin for UHT processed milk. The attention for the national product, maybe recognized in a known brand as a synonym of guaranteed quality and traceability, is therefore also important for UHT milk. The latter, despite being heat-treated and therefore safer from a microbiological point of view, still does not offer buyers the transparency on the indication of origin extremely sought after by consumers [33,34,60,64]. On the contrary, this consumer considered milk taste, fat content, packaging material, organic certification, nutritional value, and label information as unimportant for product choice. The simultaneous negative evaluation of taste, fat content, and nutritional value agrees with this group’s choice of skimmed and semi-skimmed milk. This result probably reflects the lack of attention of this profile towards both the product nutritional value, which is partially deteriorated in the UHT product, and taste, which is also conveyed by the fat, is consequently less perceptible in the skimmed UHT milk [96], as well as being negatively affected by ultra-high temperature treatment.

The FPc decision-making profile chose milk paying attention to product origin, especially the local origin, expiry date, high-quality certification, and on taste. This latter aspect is in line with the choice of the considered sample in this research that selecting, in contrast to UHT_c_, semi-skimmed and whole milk whose characteristics (and especially fat content) are reflected in a more perceptible taste and greater nutritional value [97,98]. A high-fat level was often associated with the choice of FP milk in different literature researches [99].

In contrast to UHT_c_, the interpretation of the importance given to the expiry date, in this case, is probably related to the poor shelf-life of the fresh product, which therefore needs to be monitored at the moment of the purchase. This assumption could be confirmed also considering the declared frequency of milk purchase by this consumer group, which is in line with the importance given to the expiration date. This latter result confirms the data relating to the study on fresh milk consumption in Italy [100], characterised by a good frequency but a low quantity per act of consumption.

Concerning the other attributes evaluation, these consumers tend to choose local branded products with high quality certification, emphasizing the perceived relationship between the local/sustainable dimension and the domestic raw milk origin [101]. This finding suggests that consumers concerned about local production perceive the higher quality of the produced milk as sustainable and safer. The choice of proximity and traditional points of purchase, in this case, is reflected in the idea that the fresh product is synonymous with the local and heritage production. 

### 3.3. MANOVA Results: Effect of the Socio-Demographic Variables and the Milk’s Type on Consumer Preferences

Our results show that UHT_c_, if compared to FPc, are characterised by: (i) a younger demographic profile, (ii) a lower average annual income, and (iii) a majority of employees with a medium-high level of education. In accordance to their answers about the purchasing motivation of UHT milk, this group seems to be inclined to choose a milk that offers many advantages of product convenience (with longer shelf-life and higher storage temperatures) [69,102]. This result is consistent with previous findings in literature: younger people with an active job are more likely to choose UHT milk [103,104], forgoing the nutritional and organoleptic quality [96,105] of the product and emphasising the utilitarian dimension (level of service, how useful or beneficial the product is) [90]. The latter characteristics of the UHT_c_ sample are also reflected in the occupation of the respondents belonging to this group: active persons with fixed timetables are closely linked to the choice orientation towards convenience food products [106]. On the other hand, this decision-making profile differs from that described for older individuals: they probably have more time for shopping and greater attention to nutritional and health product aspects [53,107]. Our results report a disparity in the average annual household income between the two considered groups, showing that the UHT_c_ has a lower mean revenue. This result is in disagreement with other studies carried out at national level [55,84] in which it emerges that household income does not play a discriminating role in defining the purchase choices of milk consumers. However, even if drinking milk on the national market has an affordable price per litre (i.e., from less than 1 euro for UHT milk to almost 3 euros for FP milk) [39], the monthly expenditure for milk could have an important incidence on the family budget, especially in households with a low income. The FPc group was represented above all by housewives and self-employed workers, characterised by older average, that bought fresh pasteurized milk several times a week, profiling a purchasing behaviour in line with the greater availability and time to spend to household shopping [108].

The MANOVA analysis was performed to investigate the influence of SD variables, simultaneously with the type of milk chosen habitually, on the individual preferences expressed to the 12 considered milk attributes. The impact of SD variables on the consumer decision-making process has been previously and widely proven in several literature researches [90,109,110,111,112]. In this research, when we analysed the variable Occupation, the categories of homemakers and unemployed persons were excluded from the MANOVA analysis due to their limited size.

Significant differences were found considering the SD variables both independently and in the combination with the type of milk. The MANOVA results are described in Tables S1 and S2 in the supplementary material, while the differences in A-RS for each milk attributes across the different SD sub-groups are reported in Table 3. Firstly, in all the conducted MANOVAs the single effect of the type of milk emerged statistically significant with a Partial η^2^ always ≥0.200. This result explains how the type of milk has a good effect in explaining more than 20% of the variance in the definition of milk consumer preferences, confirming how different product varieties and, consequently, their intrinsic, extrinsic and usage characteristics, determine different levels of preference towards the same quality attributes (Figure 1) [90]. Furthermore, even when considering the interaction between type of milk and socio-demographic variables, these were always statistically significant, except in the case of the combination with gender (λ = 0.984, *p* = 0.103), family composition (λ = 0.983, *p* = 0.059) and educational level (λ = 0.959, *p* = 0.063). In the latter cases, therefore, the individual variables exert a greater effect on the variance of individuals’ preferences than the combined variables.

In detail, firstly considering gender, the MANOVA results indicate that also in case of the single effect of the variable, the two gender groups did not display significant differences in milk preferences (Wilks’ λ = 0.984; *p* > 0.05). This result is surprising given the numerous works in which gender has emerged as a discriminating factor in the definition of individuals’ preferences [6,37,113,114]. It is likely that this result could have been influenced by the composition of our sample, typical of Italian household milk purchasers [81,82], as well as by a probable universality of choices between genders towards a common product such as milk.

On the contrary, from the MANOVA analysis emerged how the consumer preferences towards milk attributes changed significantly across the different age groups (Table 3) (λ = 0.915, F = 4.521, *p* = 0.000, Partial η^2^ = 0.065). Partial eta squared (0.065) evidenced a reasonably good effect size, indicating that age explains 6.5% of variance the consumer preferences definition. In this case, also the interaction (type of milk*age) affected significantly the consumer preferences (λ = 0.957, F = 2.220, *p* = 0.002, Partial η^2^ = 0.022). 

In particular, the assessment of expiry date and taste resulted heterogeneous into the same group between the young and the over 65 s consumer. While young people rated the use-by date as an important attribute in their choice, more mature UHT_c_ did not, perhaps since they belong to the group of shoppers who used to buy this product several times a week. As far as taste is concerned, young people in the FPc group contrasted again with older consumers, showing an attitude of choice, which can be traced back to utilitarian products. 

Also, the multivariate main effect for the family composition was significant (Table S1, supplementary material) (λ = 0.957, F = 4.437, *p* = 0.000, Partial η^2^ = 0.059), but not its interaction with the type of milk. This result indicates the importance of the presence of children in the family in the preferences definition, regardless of the type of product chosen. Considering the differences of A-RS between the two groups (Table 3) the presence of children affects the perception of price, organic certification, fat content, taste, and expiration date.

Regarding the occupation, both the main effect of the variable (λ = 0.858, F= 2.556, *p* = 0.000, Partial η^2^ =0.025) and its interaction with the type of milk habitually chosen (λ = 0.922, F= 1.351, *p* = 0.031, Partial η^2^ = 0.014) were statistically significant. However, the occupation variable, considering both the main effect and the interaction, shows a low effect size in the preferences definition. As emerged from the results described in Table 3, the A-RS appeared into the same consumer group different considering the taste (with a negative perception for the employed consumers), and brand for the FPc. However, in the UHT_c_ group, individuals with different employment express different preferences for the fat content, expiration date (negative only for retired) taste and local production. These results indicate how the different employment affect also into the same group the preferences towards the same attribute.

Only the main effect of the education level (λ = 0.921, F= 2.772, *p* = 0.000, Partial η^2^ = 0.027) result significant in the definition of milk consumer preferences, controversially to its interaction effect with the type of milk (λ = 0.959, *p* = 0.063). Into the same group of FPc the only attribute for which different sign of A-RS emerged is the price (most important for people with the lower educational level). Also in the UHT_c_, the primary school certified distinguish from the other individuals for the positive perception of the fat content in the milk.

Finally, the main effect of the average annual income of the family were significant for consumer preferences during milk choice (λ = 0.865, F = 3.648, *p* = 0.000, Partial η^2^ = 0.036), and in combination with the type of milk chosen (λ = 0.935, F = 1.689, *p* = 0.003, Partial η^2^ = 0.017). The average annual income has the most visible variability of the A-RS into the same consumer group (Table 3). In particular, it is on the price, fat content, and taste that this characteristic determines positive and negative preference index values. It is worth noting that the evaluation of price during the choice assumes greater importance in the more affluent consumers. At first sight, this result may seem unexpected, but a positive evaluation of the product price could translate into a positive price-quality interpretation, which has often been found in various consumer studies [72,115,116].

Considering the effect size of the single SD variable, the age and family composition are much impactful than the other SD variables: this result explain how the age and the presence/absence of children in the family explain the variation of the consumer preferences than the amount of variance accounted by the respondent’s occupation, educational level and average income. Consequently, the age of and the family size could be seen as a segregating variable relating to milk purchasing behaviour and preferences. At the same time, considering the interaction effect with the type of milk, that with the age showed the higher effect size (Partial η^2^ = 0.022) with respect to the other considered combinations.

## 4. Conclusions

In conclusion, the present research contributes to enriching the lacking literature on milk consumers in terms of profiling FP and UHT consumer preferences and characteristics, exploiting the advantages of the BWS methodology. Although the two consumer groups were not dissimilar in terms of milk choice motivations, place of purchase, and milk consumption habits, the analyses of SD characteristics, milk purchase, and consumption habits of the two groups show that the type of milk mainly affected consumer preferences (together with some individuals’ features). Considering the SD variables, it appears that the attributes valued with the greatest variability within the same group according to socio-demographic characteristics are price, fat content, and taste. While convenience still seems to be the primary requirement sought in UHT milk, given the attention that this group of consumers has expressed towards the origin of the product, the guarantee of the product origin should probably be part of future work to improve the transparency of information on the label. A more precise indication of origin for domestic products could improve the competitiveness of the Italian production, also aiming at an added value on the market. Another way of adding value to the milk could be the packaging developing that, in our study, emerges always as unimportant to the consumer. In the case of fresh milk, the importance given to traditional milk attributes, related to local origin, high nutritional value, and good product taste, highlight the consumers’ desire to return to genuine traditional products that emphasise the link with the territory (and for new generations as well). The attention of fresh milk consumers towards the local product implies greater awareness also to the social dimension of the product sustainability.

This research provides valuable results to the production, breeding, and processing sector to orient production choices, milk differentiation on the market, and communication in line with the needs of new consumption profiles and orientations. Our results highlight the importance of studying demand, especially for a product such as milk, which, in its uniqueness, should be enhanced through different market differentiation factors defined according to the consumer’s targets need.

The combination of the study of individuals’ preferences with the analysis of the effect of socio-demographic characteristics on the definition of preferences lays the basis for the creation of communication campaigns for the different consumption targets, for example emphasising key aspects for younger and mature consumers considering the two product lines of the conventional drinking milk.

Among the limits of this research, we can recognize the lack of inclusion of other variables (such as those relating to purchasing and consumption habits) in the statistical processing. However, this choice was made to narrow down the objectives by focusing on the effect of socio-demographic characteristics and the type of milk on the preferences of individuals. Furthermore, although the BWS methodology has many advantages and overcomes the limitations of traditional discrete choice analysis methodologies, according to the theories expressed by some authors [117], in our research it is the limited size of the two samples and the nature of the items used as a whole which could represent a further limitation. It has been seen, in fact, that the common perception towards an attribute evaluated “positively” or “negatively” in the common imagination (e.g., price vs. origin) influences the choices of individuals, creating a problem of BWS measures that reflect positions of absolute and relative preferences.

However, these limitations certainly lay the basis for the development of further future researches aimed at an extension of the sample, both numerically, considering other geographical areas, and at the reprogramming of the experimental design using different choice items to allow a comparison with the presented results.

In addition, further studies that include an exploration of variables directly attributable to the definition of people’s lifestyles (beliefs, norms and values related to individuals’ concern for health, environment, etc.) could help complete this research for profiling individuals and defining purchasing planned behaviour.

## Figures and Tables

**Table 1:** Questionnaire structure.

Sections	Required Information (Questions)	Possible Answers	
(1) Closed-ended questions	1a. Gender	Male, female	
	1b. Age	Open answer	
	1c. Occupation	Housekeeper, unemployed, employed, self-Employed, retired, student	
	1d. Educational level	Primary school, lower secondary school, upper secondary school, master’s degree	
	1e. Average income of the family	<25,000, 25,000–40,000, 40,000–60,000, >60,000 (€/year)	
	1f. Family size	1 member, 2 members, 3 members, 4 members, equal or more than 5 members	
	1g. Nationality	Open answer	
(2) CATA questions (*)/Closed-ended questions	2a. What type of cow’s milk do you buy?	Fresh pasteurized, UHT, both	
	2b. Why do you choose UHT? (*)	Lower cost, doesn’t clutter the fridge, I do not buy daily/weekly, I buy monthly, longer minimum storage time	
	2c. Do you choose: (*)	Whole milk, partially skimmed milk, skimmed milk	
	2d. Where do you usually buy it? (*)	Hyper/supermarket, open-air market, directly to the producer, discount, convenience store	
	2e. How many times a week do you buy milk?	Less than one, one to two times, three to five times, more than five times	
	2f. At what times of the day do you consume milk? (*)	During the meals, at breakfast, at snack time, in the evening	
	2g. Why do you buy milk? (*)	It is tasty, it is good for us, I use it in the diet, I consider it indispensable	
(3) Best-Worst scheme implementation	Example of BWS question: Indicate the most important (BEST) and the least important (WORST) attributes during the milk choice:		
	Most important (BEST)	Milk attributes	Least Important (WORST)
	x	Price	
		Local origin	
		Nutritional value	
		Packaging materials	x

(*) indicates the CATA (check-all-that-apply) questions. UHT, ultra-high temperature; BWS, Best-Worst scaling.

**Table 2:** Socio-demographic profiles of FP (*n* = 677) and UHT (*n* = 430) milk consumers (*n* = 1107).

Individual Socio-Demographic Characteristics	Total Sample	FP_c_	UHT_c_	Chi-Square	*p*-Value ^1^
Gender				0.265	0.607
Women	68%	67%	69%		
Men	32%	33%	31%		
Age groups				9.879	0.007 **
18–45	36%	33%	42%		
46–65	36%	32%	38%		
>65	28%	25%	30%		
Family composition				4.50	0.034 *
With children	43%	46%	39%		
Without children	57%	54%	61%		
Occupation				27.013	<0.001 ***
Homemaker	7%	10%	4%		
Unemployed	4%	5%	4%		
Employed	43%	38%	45%		
Self-employed	10%	12%	9%		
Retired	30%	31%	30%		
Student	6%	4%	9%		
Educational level				8.551	0.036 *
Primary school	7%	6%	9%		
Lower secondary school	20%	18%	21%		
Upper secondary school	46%	50%	42%		
Master’s degree	27%	26%	28%		
Average annual income of the family (€/year)				26.972	<0.001 ***
Lower than 25,000	27%	23%	33%		
From 25,000 to 40,000	35%	34%	36%		
More than 40,000	17%	18%	17%		
Do not answer	21%	25%	15%		

^1^ The *p*-value refers to the statistical significance level: *** *p* < 0.001, ** *p* < 0.01, * *p* < 0.05. The absence of asterisks indicates the non-significance of the value. FP, fresh pasteurized; UHT, ultra-high temperature.

**Table 3:** Differences of the average raw scores (preferences level) for each milk attributes considering the effect of the type of milk chosen (fresh pasteurised, FP; ultra-high temperature, UHT treated milk) and the individuals’ socio-demographic (SD) characteristics simultaneously.

Independent Variables		Dependent Variables											
Milk Type	SD Variables	Price	Organic Certification	Fat Content	Expiration Date	Taste	Packaging Material	HQ Certification	Local Origin	Country of Origin	Brand	Informationon the Label	Nutritional Value
FP	Women	−0.136	−1.231	−1.567	1.175	−0.113	−1.942	1.176	1.441	2.222	0.473	−0.804	−0.694
	Men	−0.157	−1.219	−1.540	1.050	0.000	−1.671	0.981	1.375	2.011	0.623	−0.817	−0.635
UHT	Women	0.606	−1.684	−0.473	0.648	−0.445	−1.041	0.566	0.410	1.685	0.877	−0.727	−0.423
	Men	0.547	−1.711	−0.053	0.458	−1.019	−0.845	0.472	0.337	1.861	1.358	−0.880	−0.525
FP	18–45	−0.039	0.200	−1.298	1.188	0.122	−1.758	1.079	1.238	1.865	0.395	−0.813	−0.732
	46–65	−0.234	0.078	−1.816	1.114	−0.126	−2.083	1.243	1.608	2.345	0.390	−0.675	−0.658
	>65	−0.140	0.144	−1.514	1.100	−0.238	−1.659	0.977	1.375	2.227	0.842	−0.980	−0.632
UHT	18–45	0.495	−1.255	−0.312	1.093	−0.024	−1.315	0.526	0.266	1.216	0.307	−0.582	−0.415
	46–65	0.673	−1.541	−0.655	0.492	−0.644	−0.877	0.642	0.518	1.693	0.963	−0.742	−0.522
	>65	0.634	−2.575	−0.014	−0.101	−1.561	−0.567	0.428	0.424	2.635	2.258	−1.123	−0.438
FP	Self-employed	−0.379	−1.099	−1.613	1.280	−0.175	−1.887	1.353	1.324	1.987	0.753	−0.825	−0.707
	Employed	−0.029	−1.128	−1.662	1.065	−0.033	−1.843	1.244	1.311	2.024	0.474	−0.793	−0.678
	Retired	−0.050	−1.507	−1.464	0.982	−0.355	−1.672	0.911	1.565	2.332	0.971	−0.963	−0.615
	Student	−0.354	−1.258	−0.776	1.680	0.811	−2.385	0.603	1.431	1.983	−0.412	−0.591	−0.823
UHT	Self-employed	0.101	−1.571	0.896	0.884	0.028	−1.183	0.259	0.676	2.192	0.866	−0.696	−0.279
	Employed	0.528	−1.340	−0.725	0.734	−0.322	−1.260	0.648	−0.036	1.108	0.490	−0.607	−0.386
	Retired	0.635	−2.615	0.077	−0.117	−1.590	−0.551	0.377	0.169	0.991	0.171	−1.130	−0.480
	Student	0.726	−1.286	0.160	1.585	0.184	−1.464	0.438	0.401	2.602	2.390	−0.390	−0.540
FP	Primary school	0.105	−1.817	−0.780	0.782	−0.767	−1.117	0.819	0.920	1.952	1.782	−1.111	−0.767
	Lower secondary school	−0.050	−1.769	−1.260	0.958	−0.164	−1.708	0.918	1.079	2.234	1.109	−0.825	−0.522
	Upper secondary school	−0.193	−1.184	−1.703	1.269	−0.107	−1.845	1.217	1.529	2.140	0.493	−0.881	−0.736
	Master’s degree	−0.167	−0.791	−1.668	1.080	0.203	−2.142	1.113	1.562	2.167	−0.127	−0.586	−0.644
UHT	Primary school	0.643	−2.905	0.352	0.065	−1.745	−0.245	0.306	0.345	2.598	2.227	−1.099	−0.543
	Lower secondary school	0.616	−1.889	−0.291	0.469	−0.675	−0.819	0.536	0.543	1.831	1.035	−0.831	−0.526
	Upper secondary school	0.589	−1.599	−0.658	0.612	−0.456	−1.132	0.663	0.358	1.716	0.995	−0.684	−0.404
	Master’s degree	0.546	−1.287	−0.128	0.818	−0.474	−1.111	0.422	0.327	1.425	0.676	−0.762	−0.449
FP	Lower than 25000	−0.059	−1.454	−1.085	1.168	0.029	−1.556	0.848	1.320	1.955	0.549	−0.864	−0.850
	From 25000 to 40000	−0.248	−0.565	−2.195	0.959	0.051	−2.186	1.503	1.579	2.242	0.177	−0.712	−0.605
	More than 40000	0.163	−1.663	−1.588	0.854	−0.391	−1.665	1.069	1.334	2.294	1.131	−0.942	−0.596
	Do not answer	0.088	−1.534	−1.502	1.038	−0.179	−1.895	1.239	1.248	2.326	0.827	−0.986	−0.670
UHT	Lower than 25000	0.645	−1.897	0.144	0.816	−0.446	−0.983	0.364	0.373	1.513	0.811	−0.817	−0.525
	From 25000 to 40000	0.556	−2.043	−1.118	0.233	−1.191	−0.295	0.688	0.817	2.538	1.389	−1.061	−0.513
	More than 40000	0.684	−1.711	−0.448	0.439	−0.600	−1.319	0.674	0.351	1.840	1.190	−0.768	−0.333
	Do not answer	1.079	−1.811	−0.871	−0.307	−2.010	−0.343	0.684	0.270	2.411	2.371	−0.828	−0.644
FP	Without children	−0.151	−1.249	1.451	−0.739	−0.203	−1.718	1.044	1.451	2.244	0.748	−0.895	−0.739
	With children	−0.133	−1.200	−1.548	1.253	0.073	−2.014	1.192	1.382	2.045	0.254	−0.706	−0.599
UHT	Without children	0.688	−1.848	−0.255	0.343	−1.031	−0.813	0.576	0.371	1.914	1.374	−0.864	−0.455
	With children	0.433	−1.452	−0.477	0.968	0.004	−1.238	0.476	0.412	1.471	0.491	−0.636	−0.454

SD, socio-demographic (variables); FP, fresh pasteurized; UHT, ultra-high temperature.

## Data Availability

The data are available on request from the authors.

## Supplementary Materials

The following supporting information can be downloaded at: https://www.mdpi.com/article/10.3390/foods11010077/s1. Table S1: Result of multivariate analyses of variance (MANOVA) of the main effect of the variables gender, age and family composition and their interaction effect with the type of milk variable; Table S2: Result of multivariate analyses of variance (MANOVA) of the main effect of the variables occupation, educational level and annual average income of the family and their interaction effect with the type of milk variable.
