# Supplementary material for: Which Factors Drive Consumer Decisions during Milk Purchase? New Individuals’ Profiles Considering Fresh Pasteurized and UHT Treated Milk
Source: Foods. 2021 Dec 29;11(1):77. doi: 10.3390/foods11010077 (PMC8750682; doi:10.3390/foods11010077)
Supplement: Supplementary file 1 [file foods-11-00077-s001.zip › foods-1517011-supplementary.pdf]

foods

## Supplementary material

**Table S1.** Result of multivariate analyses of variance (MANOVA) of the main effect of the variables gender, age and family composition and their interaction effect with the type of milk variable.

| Dependent variable | Independent variable | df | F | Partial η2 | Independent variable | df | F | Partial η2 | Independent variable | df | F | Partial η2 |
|---|---|---|---|---|---|---|---|---|---|---|---|---|
| Price | Gender | 1 | 0.172 | 0.000 | Age | 2 | 0.030 | 0.000 | Family composition | 1 | 1.669 | 0.002 |
| Organic certification | | 1 | 0.003 | 0.000 | | 2 | 13.046*** | 0.023 | | 1 | 3.293 | 0.003 |
| Fat content | | 1 | 2.139 | 0.002 | | 2 | 4.460* | 0.008 | | 1 | 0.492 | 0.000 |
| Expiration date | | 1 | 1.845 | 0.002 | | 2 | 11.628*** | 0.021 | | 1 | 14.930*** | 0.013 |
| Taste | | 1 | 3.935* | 0.004 | | 2 | 25.644*** | 0.045 | | 1 | 36.736*** | 0.032 |
| Packaging material | | 1 | 4.742* | 0.004 | | 2 | 6.468*** | 0.012 | | 1 | 12.716*** | 0.011 |
| High quality certification | | 1 | 2.620 | 0.002 | | 2 | 2.568 | 0.005 | | 1 | 0.081 | 0.000 |
| Local origin | | 1 | 0.572 | 0.001 | | 2 | 4.699** | 0.008 | | 1 | 0.026 | .0000 |
| Country of origin | | 1 | 0.020 | 0.000 | | 2 | 19.514*** | 0.034 | | 1 | 7.309** | 0.007 |
| Brand | | 1 | 3.843* | 0.003 | | 2 | 21.788*** | 0.038 | | 1 | 20.769*** | 0.018 |
| Information on the label | | 1 | 1.093 | 0.001 | | 2 | 8.943*** | 0.016 | | 1 | 7.742* | 0.007 |
| Nutritional value | | 1 | 0.085 | 0.000 | | 2 | 0.207 | 0.000 | | 1 | 1.030 | 0.001 |
| Price | Type of milk*gender | 1 | 0.038 | 0.000 | Type of milk*age | 2 | 1.586 | 0.003 | Type of milk*family compositition | 1 | 2.219 | 0.002 |
| Organic certification | | 1 | 0.022 | 0.000 | | 2 | 8.220*** | 0.015 | | 1 | 2.014 | 0.002 |
| Fat content | | 1 | 1.656 | 0.001 | | 2 | 1.046 | 0.002 | | 1 | 0.690 | 0.001 |
| Expiration date | | 1 | 0.078 | 0.000 | | 2 | 8.544*** | 0.015 | | 1 | 3.442 | 0.003 |
| Taste | | 1 | 8.781** | 0.008 | | 2 | 10.004*** | 0.018 | | 1 | 12.324*** | 0.011 |
| Packaging material | | 1 | 0.122 | 0.000 | | 2 | 6.167*** | 0.011 | | 1 | 0.408 | 0.000 |
| High quality certification | | 1 | 0.316 | 0.000 | | 2 | 0.040 | 0.000 | | 1 | 2.126 | 0.002 |
| Local origin | | 1 | 0.002 | 0.000 | | 2 | 0.247 | 0.000 | | 1 | 0.392 | 0.000 |
| Country of origin | | 1 | 2.382 | 0.002 | | 2 | 8.420*** | 0.015 | | 1 | 1.053 | 0.001 |
| Brand | | 1 | 1.049 | 0.001 | | 2 | 8.334*** | 0.015 | | 1 | 1.663 | 0.002 |
| Information on the label | | 1 | 0.781 | 0.001 | | 2 | 2.487 | 0.004 | | 1 | 0.066 | 0.000 |
| Nutritional value | | 1 | 1.215 | 0.001 | | 2 | 0.666 | 0.001 | | 1 | 1.017 | 0.001 |

The significant p-value: ***<0.001; **<0.01; *<0.05. The absence of asterisks indicates the non-significance of the value.

**Table S2.** Result of multivariate analyses of variance (MANOVA) of the main effect of the variables occupation, educational level and annual average income of the family and their interaction effect with the type of milk variable.

| Dependent variable | Independent variable | df | F | Partial η2 | Independent variable | df | F | Partial η2 | Independent variable | df | F | Partial η2 |
|---|---|---|---|---|---|---|---|---|---|---|---|---|
| Price | Occupation | 3 | 0.898 | 0.005 | Educational level | 3 | 0.494 | 0.001 | Average income | 4 | 13.207*** | 0.046 |
| Organic certification | | 3 | 7.731*** | 0.041 | | 3 | 13.072*** | 0.034 | | 4 | 9.472*** | 0.033 |
| Fat content | | 3 | 3.208** | 0.017 | | 3 | 4.729* | 0.013 | | 4 | 5.100*** | 0.018 |
| Expiration date | | 3 | 5.865*** | 0.031 | | 3 | 2.699* | 0.007 | | 4 | 8.820*** | 0.031 |
| Taste | | 3 | 11.095*** | 0.057 | | 3 | 8.958*** | 0.024 | | 4 | 11.964*** | 0.042 |
| Packaging material | | 3 | 3.665*** | 0.020 | | 3 | 8.100*** | 0.022 | | 4 | 2.272 | 0.008 |
| High quality certification | | 3 | 2.389** | 0.013 | | 3 | 2.671* | 0.007 | | 4 | 2.772* | 0.010 |
| Local origin | | 3 | 0.899 | 0.005 | | 3 | 1.509 | 0.004 | | 4 | 1.753 | 0.006 |
| Country of origin | | 3 | 7.173*** | 0.038 | | 3 | 1.553 | 0.004 | | 4 | 4.539** | 0.016 |
| Brand | | 3 | 12.482*** | 0.064 | | 3 | 12.215*** | 0.032 | | 4 | 14.027*** | 0.049 |
| Information on the label | | 3 | 4.189*** | 0.022 | | 3 | 2.789* | 0.008 | | 4 | 2.608* | 0.009 |
| Nutritional value | | 3 | 0.793 | 0.004 | | 3 | 0.298 | 0.001 | | 4 | 1.917 | 0.007 |
| Price | Type of milk* occupation | 3 | 0.831 | 0.005 | Type of milk*educational level | 3 | 0.197 | 0.001 | Type of milk*average income | 4 | 0.727 | 0.003 |
| Organic certification | | 3 | 3.057** | 0.017 | | 3 | 1.256 | 0.003 | | 4 | 1.730 | 0.006 |
| Fat content | | 3 | 2.293** | 0.012 | | 3 | 0.862 | 0.002 | | 4 | 0.498 | 0.002 |
| Expiration date | | 3 | 2.957** | 0.016 | | 3 | 0.842 | 0.002 | | 4 | 2.240 | 0.008 |
| Taste | | 3 | 3.410** | 0.018 | | 3 | 1.032 | 0.003 | | 4 | 6.374*** | 0.023 |
| Packaging material | | 3 | 1.524 | 0.008 | | 3 | 0.598 | 0.002 | | 4 | 7.103*** | 0.025 |
| High quality certification | | 3 | 0.754 | 0.004 | | 3 | 0.533 | 0.001 | | 4 | 1.041 | 0.004 |
| Local origin | | 3 | 2.647** | 0.014 | | 3 | 3.834* | 0.010 | | 4 | 0.337 | 0.001 |
| Country of origin | | 3 | 4.102*** | 0.022 | | 3 | 2.778* | 0.008 | | 4 | 1.385 | 0.005 |
| Brand | | 3 | 2.859*** | 0.015 | | 3 | 1.370 | 0.004 | | 4 | 2.669* | 0.010 |
| Information on the label | | 3 | 1.422 | 0.008 | | 3 | 1.485 | 0.004 | | 4 | 0.679 | 0.002 |
| Nutritional value | | 3 | 0.359 | 0.002 | | 3 | 1.148 | 0.003 | | 4 | 0.474 | 0.002 |

The significant p-value: ***<0.001; **<0.01; *<0.05. The absence of asterisks indicates the non-significance of the value.
